# Validation of a high-confidence regulatory network for gene-to-NUE phenotype in field-grown rice

**DOI:** 10.3389/fpls.2022.1006044

**Published:** 2022-11-25

**Authors:** Carly M. Shanks, Ji Huang, Chia-Yi Cheng, Hung-Jui S. Shih, Matthew D. Brooks, José M. Alvarez, Viviana Araus, Joseph Swift, Amelia Henry, Gloria M. Coruzzi

**Affiliations:** ^1^ Department of Biology, Center for Genomics and Systems Biology, New York University, New York, NY, United States; ^2^ Department of Life Science, College of Life Science, National Taiwan University, Taipei, Taiwan; ^3^ Global Change and Photosynthesis Research Unit, United States Department of Agriculture (USDA) Agricultural Research Service (ARS), Urbana, IL, United States; ^4^ Centro de Biotecnología Vegetal, Facultad de Ciencias de la Vida, Universidad Andrés Bello, Santiago, Chile; ^5^ Agencia Nacional de Investigación y Desarrollo–Millennium Science Initiative Program, Millennium Institute for Integrative Biology (iBio), Santiago, Chile; ^6^ Departamento de Genética Molecular y Microbiología, Pontificia Universidad Católica de Chile, Santiago, Chile; ^7^ Plant Biology Laboratory, The Salk Institute for Biological Studies, La Jolla, CA, United States; ^8^ Rice Breeding Innovations Platform, International Rice Research Institute, Los Baños, Laguna, Philippines

**Keywords:** rice, drought, nitrogen, gene regulatory network, network validation, NUE, GENIE3, WGCNA

## Abstract

Nitrogen (N) and Water (W) - two resources critical for crop productivity – are becoming increasingly limited in soils globally. To address this issue, we aim to uncover the gene regulatory networks (GRNs) that regulate nitrogen use efficiency (NUE) - as a function of water availability - in Oryza sativa, a staple for 3.5 billion people. In this study, we infer and validate GRNs that correlate with rice NUE phenotypes affected by N-by-W availability in the field. We did this by exploiting RNA-seq and crop phenotype data from 19 rice varieties grown in a 2x2 N-by-W matrix in the field. First, to identify gene-to-NUE field phenotypes, we analyzed these datasets using weighted gene co-expression network analysis (WGCNA). This identified two network modules ("skyblue" & "grey60") highly correlated with NUE grain yield (NUEg). Next, we focused on 90 TFs contained in these two NUEg modules and predicted their genome-wide targets using the N-and/or-W response datasets using a random forest network inference approach (GENIE3). Next, to validate the GENIE3 TF→target gene predictions, we performed Precision/Recall Analysis (AUPR) using nine datasets for three TFs validated *in planta*. This analysis sets a precision threshold of 0.31, used to "prune" the GENIE3 network for high-confidence TF→target gene edges, comprising 88 TFs and 5,716 N-and/or-W response genes. Next, we ranked these 88 TFs based on their significant influence on NUEg target genes responsive to N and/or W signaling. This resulted in a list of 18 prioritized TFs that regulate 551 NUEg target genes responsive to N and/or W signals. We validated the direct regulated targets of two of these candidate NUEg TFs in a plant cell-based TF assay called TARGET, for which we also had *in planta* data for comparison. Gene ontology analysis revealed that 6/18 NUEg TFs - OsbZIP23 (LOC_Os02g52780), Oshox22 (LOC_Os04g45810), LOB39 (LOC_Os03g41330), Oshox13 (LOC_Os03g08960), LOC_Os11g38870, and LOC_Os06g14670 - regulate genes annotated for N and/or W signaling. Our results show that OsbZIP23 and Oshox22, known regulators of drought tolerance, also coordinate W-responses with NUEg. This validated network can aid in developing/breeding rice with improved yield on marginal, low N-input, drought-prone soils.

## Introduction

Nitrogen (N) and water (W) are essential resources for plant productivity that are becoming increasingly limited in marginal soils world-wide (Gibbs and Salmon, 2015; Hsieh et al., 2018). Moreover, applications of N and W in agriculture are costly resources to society (Williamson, 2011; Keeler et al., 2016; D'Odorico et al., 2020). Most studies in major crops like rice, examine the effects of N and drought separately (Anantha et al., 2016; Li et al., 2017; Volante et al., 2017; Zhao et al., 2017). More recently, studies that examine how the interaction between N and W availability affects rice phenotypes and gene regulation have been examined (Swift et al., 2019; Araus et al., 2020; Plett et al., 2020; Sevanthi et al., 2021).

Several studies have shown that genes critical to N-uptake, sensing and metabolism have been associated with a drought phenotype. For example, NRT1.1/CHL1/NPF6.3 the a dual-affinity nitrate transporter (Liu et al., 1999) is expressed in the guard cells in Arabidopsis. Moreover, *nrt1.1/chl1* mutant is more drought tolerant compared to wild-type. The loss of NRT1.1/CHL1 reduced the stomatal opening and transpiration rates which contribute to its drought-tolerant phenotype (Guo et al., 2003). Next, mutants in nitrate reductase in both Arabidopsis (NIA1 and NIA2) and rice (OsNR1.2) exhibit a drought-tolerant phenotype with reduced water loss (Lozano-Juste and Leon, 2010; Han et al., 2022). Transcription factors (TFs) are also at the center of N-by-W response. NLP7 is a master regulator of nitrogen signaling in Arabidopsis (Alvarez et al., 2020). The *nlp7* mutant shows drought resistant phenotype, similar to *nrt1.1/chl1* (Castaings et al., 2009). Putting these findings together, it has been hypothesized that NLP7 regulates NRT1.1/CHL1 expression in guard cells and further controls stomatal opening and hence drought tolerance. Another TF in rice, drought and salt tolerance (DST), also bridges between N-assimilation and stomata movement that provides a path to crop improvement under marginal soil (lowN-lowW) (Han et al., 2022).

**Figure 1:**
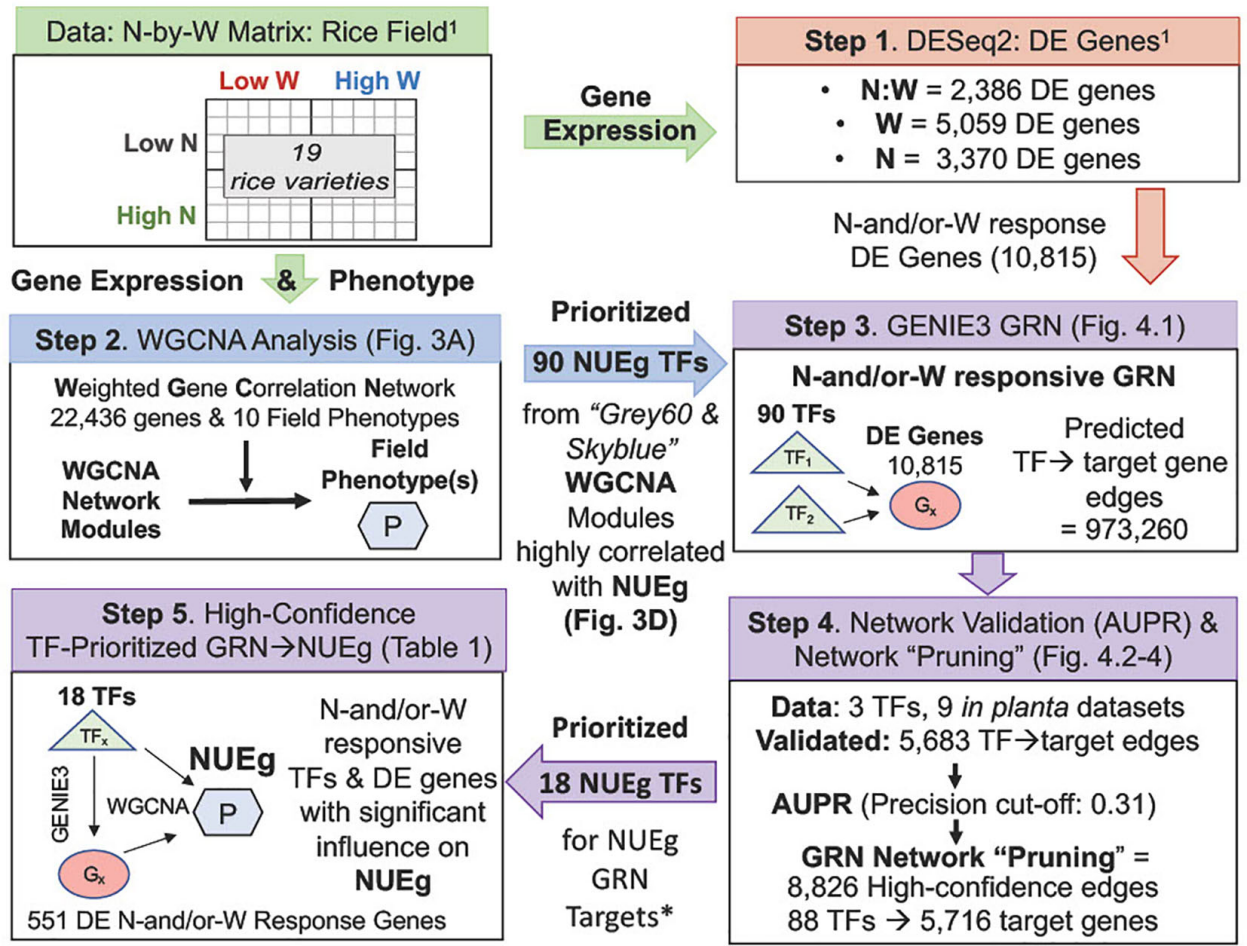
Flow-chart for generation of a high-confidence GRN of TF→target gene→NUEg phenotype from rice field data. Gene expression and phenotype data from field grown rice used to generate the WGCNA modules and GRN were obtained from 19 rice varieties of varying drought resistance, grown under a 2x2 N-by-W matrix with four combinations of N and W conditions (Low vs High) from Swift et al., 2019 (Swift et al., 2019)^1^. Step 1. N-by-W matrix: RNA-seq and field phenotype data: The differentially expressed (DE) rice genes that respond exclusively to either N:W, W and N were identified using DESeq2 analysis from field gene expression data (Swift et al., 2019). Step 2. WGCNA analysis: network modules-to-phenotype: The genes/TFs highly correlated with field phenotypes were identified using the field gene expression counts of the 22,436 normalized genes and 10 field phenotypes as inputs into weighted gene co-expression network analysis (WGCNA). Step 3. GENIE3 analysis: TF→target gene predictions: The TF→target gene predictions between 90 TFs highly correlated with the NUE grain yield (NUEg) from WGCNA analysis (Step 2) and the total 10,815 N-and/or-W response genes from Swift et al., 2019 (Step 1) determined using the network inference program GENIE3 resulted in ((90 TFs*10,815 DE genes) - 90 TFs) = 973,260 edges or TF→target gene predictions) Step 4. Network validation (AUPR) and "pruning": Validation data for 3 TFs in the GENIE3 network was located using rice.connectf.org (Brooks et al., 2020), which consisted of 9 RNA-seq/ChIP-seq *in planta* datasets. This rice validation data confirmed 5,683 predicted edges for the 3 TFs was used to calculate the area under the precision/recall curve (AUPR) using automated functions in ConnecTF (Brooks et al., 2020). This AUPR was then used to select a precision cut-off and "prune" the network for high-confidence edges of the GENIE3 gene regulatory network (GRN), again using automated functions in ConnecTF. The "pruned" GENIE3 network consists of 8,826 high-confidence edge predictions for 88 TFs and 5,716 genes linked to the NUEg phenotype from WGCNA. Step 5. High-confidence GRN: There are 18/88 TFs in the pruned network that regulated a significant number of the genes highly correlated with NUEg as identified in the WGCNA modules, for a total of 551 DE N-and/or-W Response Genes (Step 2).*See **Table 1** and **Supplementary Figure 3** for TF prioritization results and pipeline.

On the genome-wide level, our current manuscript explores on the gene regulatory networks (GRN) involved in N-by-W interactions by mining the N-by-W response RNA-seq and phenotype dataset from field grown rice (Swift et al., 2019). In our previous Swift et al 2019 study, we used linear models to discover that N-by-W signaling (N/W, molarity and/or NxW synergistic interactions) significantly correlate with rice field phenotypes, compared to genes that respond *only* to W-dose or N-moles (Swift et al., 2019). That dataset – which we use in our current analysis includes transcriptomic and phenotypic data for 19 rice varieties that vary in their drought and N-response. These 19 rice varieties were treated in a 2x2 N-by-W matrix of two N-doses (fertilized vs. without N) and W-doses (high vs. low water) in field experiments conducted at the International Rice Research Institute (IRRI) in the Philippines (Swift et al., 2019) (**Figure 1**). While our Swift et al., 2019 study determined the importance of the N-by-W gene responses (e.g., N/W and NxW) to phenotypic field outcomes in rice, the goal of our present study is to determine the GRNs underlying TF→target gene→phenotype interactions that correlate with NUE phenotypes in the rice N-by-W field study.

To develop sustainable agricultural solutions to feed a growing population, in this study we exploit a systems biology approach to uncover and validate the gene regulatory networks (GRNs) by which rice (*Oryza sativa*) plants sense and respond to the combination of N- and W- availability to promote crop productivity. To this end, we connected gene-to-NUE phenotype using weighted gene correlation analysis (WGCNA) (Langfelder and Horvath, 2008). Next, for the target genes that correlate with NUE phenotypes, we identified TF-to-target gene relationships in a gene regulatory network (GRN) using GENIE3 (Huynh-Thu et al., 2010). We then validated the TF-to-target gene network predictions via precision/recall (AUPR) analysis using validated TF-target gene data obtained *in planta* using the ConnecTF platform (https://rice.connectf.org). Additionally, we applied the plant cell-based Transient Assay Reporting Genome-wide Effects of Transcription factors (TARGET) system (Bargmann et al., 2013; Brooks et al., 2019), which we adapted in rice to validate the high-confidence TF-to-gene network for the N-by-W response genes whose expression level correlate with NUE.

Overall, we identified six TFs that regulate genes involved in both N and/or W signaling: OsbZIP23 (LOC_Os02g52780), Oshox22 (LOC_Os04g45810), LOB39 (LOC_Os03g41330), Oshox13 (LOC_Os03g08960), LOC_Os11g38870, LOC_Os06g14670. Two of these TFs are known regulators of drought tolerance - OsbZIP23 and Oshox22 – (Xiang et al., 2008; Zhang et al., 2012; Dey et al., 2016; Zong et al., 2016). Our present study shows that these TFs involved in drought responses are also responsive to N-by-W interactions. Moreover, we show that these six TFs control N-and/or-W response genes that correlate with NUEg. This information can now be applied to develop/breed rice plants with improved yield, on marginal, low N-input, drought-prone soils and on fields where water and N are limited due to climate change.

## Materials and methods

### Source of N-by-W response data (transcriptome and phenotype) for 19 rice varieties

Field phenotypic data collection and conditions for 19 rice varieties (Indica and Japonica) can be found in Swift et al., 2019 (Swift et al., 2019). The details of the treatments are in Swift et al., 2019, but as an overview: For the +N treatment, 150 kg/ha dose of (NH4)2SO4 was applied at 23 days after sowing (DAS). The -N treatment had no addition of fertilizer. Plants in the -W condition were covered from rain with a rainout shelter (intermittent watering was applied to ensure growth), while plants in the +W condition received rainfall and normal watering. Water-use-efficiency (WUE) was determined from leaves with carbon isotope discrimination as outlined in Swift et al., 2019 (Swift et al., 2019). The nitrogen usage data was calculated using the Kjeldahl N (KJ N) method which determined the nitrogen content from 1 gram of leaf samples. The total KJ N is determined as in (Bremner and Mulvaney, 1982; Bremner, 1996) by converting organic nitrogen forms to NH_4_^3+^ and then measuring the concentration. To calculate N-uptake, we used the Kjeldahl N percent (KJ N%) and vegetative shoot dry weight (SDW) measurements from Swift et al., 2019 collected from leaf samples. We then used the N uptake measurement to calculate NUEg and NUE biomass (NUEb).


N uptake (g/m2)=(KJ N%∗SDWg/plant)∗plants/m2

$$NUEg=\frac{Grain\;yield\;g/m2}{N\;uptake\;g/m2}$$

$$NUEb=\frac{Biomass\;g/m2}{N\;uptake\;g/m2}$$

The field transcriptomic data consisted of 19 rice varieties (*Indica* and *Japonica*) of varying drought tolerant phenotypes, grown under four N-by-W treatment conditions, with three replicate leaf samples for RNA-seq for a total of 228 RNA-seq samples. Expression counts for 228 RNA-seq samples were normalized with the DESeq2 package (Love et al., 2014). The TFs and TF families from the N-and/or-W DE gene lists were identified based on the Plant Transcription Factor Database v4.0 categorization (Jin et al., 2017). See data availability in Swift et al., 2019 (Swift et al., 2019) for source phenotypes and transcriptome data.

### Potential index (I_PO_) calculation of NUE under low vs. high N and W conditions

To compare NUEg among the 19 rice varieties, we calculated the potential index (I_PO_) as similar to Ndiaye et al, 2019 (Ndiaye et al., 2019). For the calculation, each variety's NUEg was compared with the conditional average, using the formula below.


$$I_{PO}\;=\;\frac{Y_{ij}\;-\;\underline{Y_{j}}\;\;}{\underline{Y_{j}}}$$

**Figure 2:**
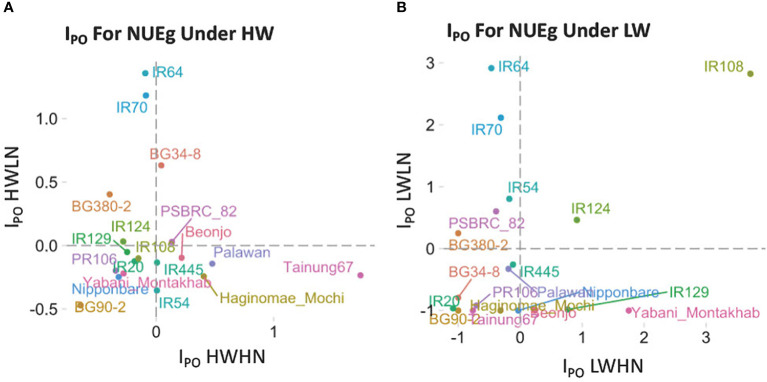
The NUEg phenotype for 19 rice varieties measured under four N-by-W conditions. We used the Potential index (I_PO_) (Ndiaye et al., 2019) on 19 rice varieties which differ in their drought resistance to assess the NUEg values under **(A)** high water and **(B)** low water conditions with varying N-doses. **(A)** DHWHN, high-W/high-N; HWLN, high-W/low N; **(B)** LWHN, low-W/high N; LWLN, low-W/low-N.

The I_PO_ is the potential index of variety *i*; *Y*_*ij*_ is the NUEg of variety *i* for the condition *j* where *j* is HWHN, HWLN, LWHN or LWLN; $\underline{Y_{j}}$ is the conditional mean of all 19 varieties under condition *j*. The I_PO_ is a relative value that shows the increase or decrease of a specific variety's NUEg, over the mean values. An I_PO_ > 0 indicates better NUEg, whereas I_PO_< 0 indicates worse NUEg (**Figure 2**). The NUEg phenotype data was downloaded from Swift et al, 2019 (Swift et al., 2019).

### WGCNA analysis: Gene-to-field phenotype correlation

**Figure 3:**
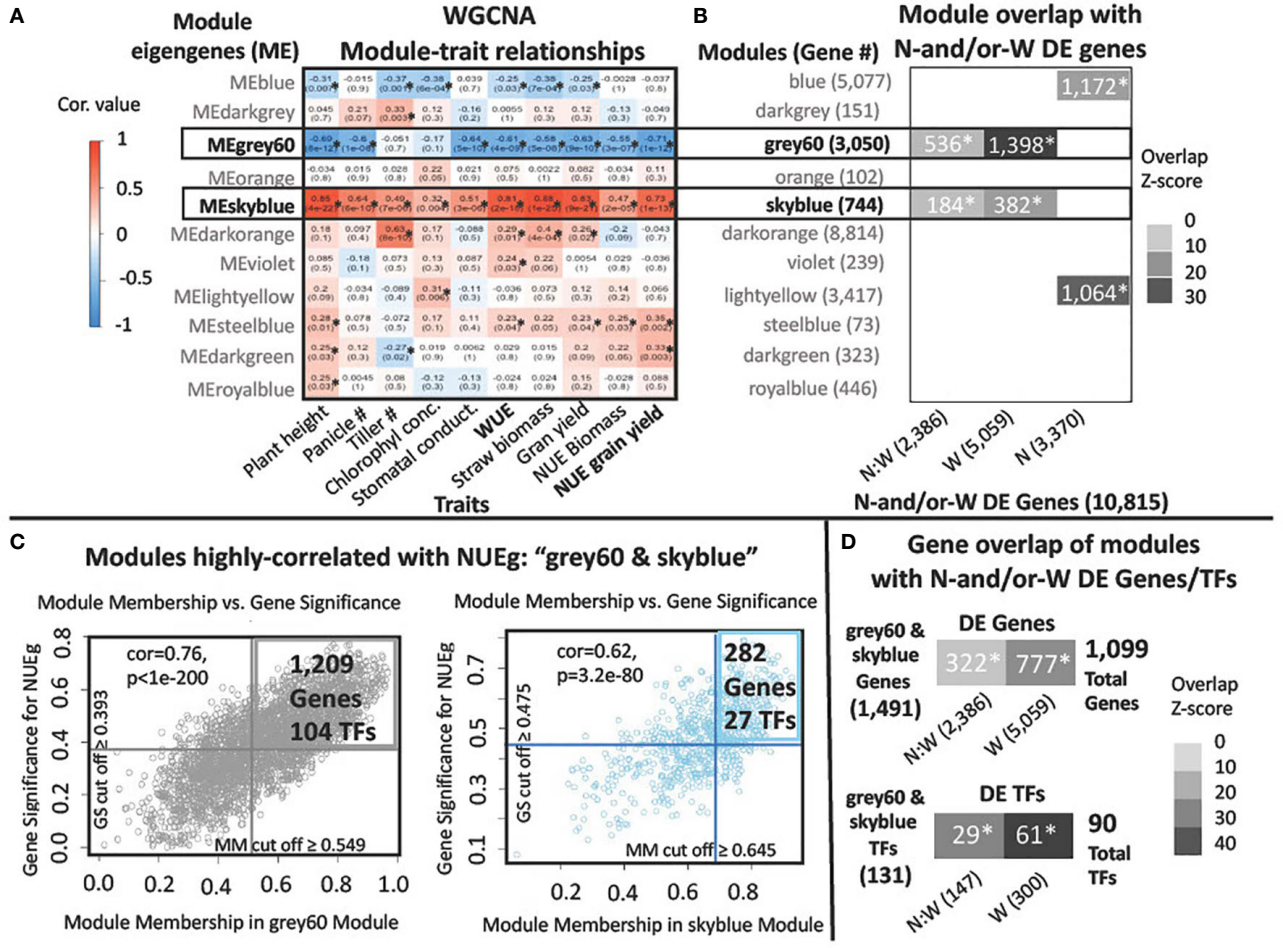
WGCNA modules named "grey60" and "skyblue" are highly correlated with NUEg in field grown rice. **(A)** Heatmap of the correlation values for the Module Eigengene (ME) values with field phenotypes from WGCNA. Red and blue colors note positive and negative correlation, respectively, for the ME for each module of co-expressed genes. Modules significantly associated with traits have a p value< 0.05, denoted by an asterisk*. **(B, D)** N-and/or-W DE genes and TFs for N:W, W and N -response genes derived from ANOVA analysis in Swift et al., 2019 (Swift et al., 2019). Heatmap of the Z-score for each overlap (Z-score ≥ 10). The p-value< 0.001 is denoted with an asterisk*. Z-score and p-values were calculated using the Genesect function in VirtualPlant 1.3 (Katari et al., 2010). **(B)** Significance of intersection between the genes in each co-expression module from WGCNA (**Supplementary Data 1**) and the N, W, and N:W DE genes, identified using Genesect function in VirtualPlant 1.3. **(C)** Scatterplots of the WGCNA Gene Significance (GS) for NUEg, versus the Module Membership (MM) for the grey60 and skyblue modules exhibit a significant correlation p-value< 0.001 with NUEg. The genes with a GS and MM cut-off scores above the average score for the genes in each module were selected for further analysis (1,209 grey60 + 282 skyblue genes = 1,491 genes). **(D)** Significance of gene intersection (using Genesect) between the union of the genes and TFs with an above-average GS and MM score from the WGCNA grey60 and skyblue modules (grey60&skyblue) and the N:W, W, or N- responsive DE genes. Union of the genes in grey60 and skyblue modules: N-and/or-W response DE TFs (29 + 61 = 90 total) used for GENIE3 network analysis and N-and/or-W response DE genes (322 + 777 = 1,099 total) used to prioritize TFs from the pruned GENIE3 network (**Supplementary Data 2**).

The normalized counts files for each treatment and genotype were averaged as inputs into WGCNA to match the averaged field phenotypes for each biological replicate. This resulted in 76 transcriptomic and phenotypic values (19 varieties and 4 treatments) as inputs into WGCNA. The transcriptome counts file consists of counts for 22,436 genes in 76 samples. The R package, WGCNA, was used to perform the weighted correlation network analysis using step-by-step network construction and module detection (Langfelder and Horvath, 2008). We selected a MEDissThres of 0.5 to combine modules correlated with each other. We averaged the absolute value of the NUEg GS, WUE GS, and module membership (MM) scores for the genes in each module to select a cut-off value for highly correlated genes. (**Figure 3C** and **Supplemental Figure 1**). Overlapping module gene lists and N-and/or-W DE gene lists were made with Venny 2.1 web tool (Oliveros, 2015). To determine the Z score and p-value of the NUEg and WUE genes that overlap with N-and/or-responsive DE gene lists, we used the Genesect function in Virtual Plant 1.3 (Katari et al., 2010) (**Figures 3B, D** and **Supplementary Figure 1**).

### GENIE3 analysis of GRNs and validation of TF→ target gene predictions by AUPR and "network pruning"

**Figure 4:**
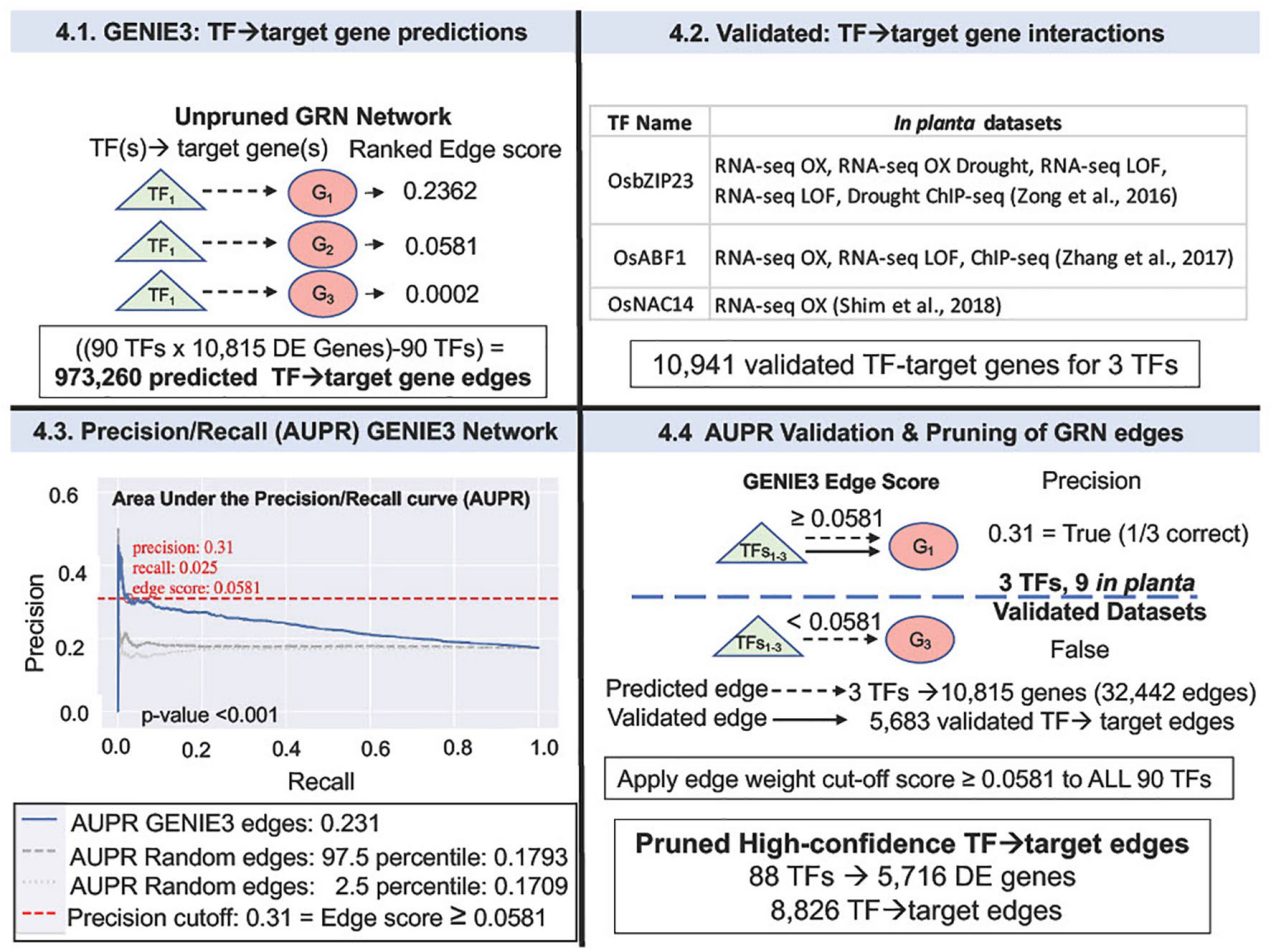
Validation of GENIE3 network using rice TF-perturbation datasets in Area Under the Precision Recall (AUPR) curve analysis. 4.1. GENIE3: The GENIE3 network ranked TF→target gene predictions for 90 N-and/or-W DE TFs (from the grey60 and the skyblue modules, **Figure 3D**), and 10,815 DE genes - each TF→target gene edge is given a weight. 4.2 The validated TF→target gene data used to "prune" the network predictions was identified using the rice TF data housed in the ConnecTF database (https://rice.connectf.org) (Brooks et al., 2020) (**Supplementary Figure 2**). Data for three TFs, OsbZIP23, OsABF1, and OsNAC14 were then used to validate the predicted GENIE3 edges with a total of 10,941 validated edges between all three TFs. 4.3. Area Under the Precision-Recall (AUPR) curve was calculated with the rice shoot *in planta* validation data for the three TFs. AUPR analysis shows that the ranking for the validated TF→target gene edges of the GENIE3 network (blue line) is significantly better (p-value<0.001, permutation test), than a set of randomly validated edges (Note: gray dashed lines are for the highest and lowest AUPR that resulted from random validated edges). A precision cut-off of 0.31 (red dashed line) was selected as the highest precision value before the curve flattens, and the "pruned" network edges were exported as an automated function in ConnecTF (Brooks et al., 2020). 4.4 The pruned GENIE3 network consists of 8,826 edges for 88 TFs and 5,716 genes that pass an edge score threshold of 0.0581. Source data of the original GENIE3 network vs. the high-confidence "pruned" GENIE3 network are supplied as **Supplemental Data 4** and **5**. Precision and Recall are calculated as in Brooks et al., 2019, 2020 (Brooks et al., 2019, 2020).

**Figure 5:**
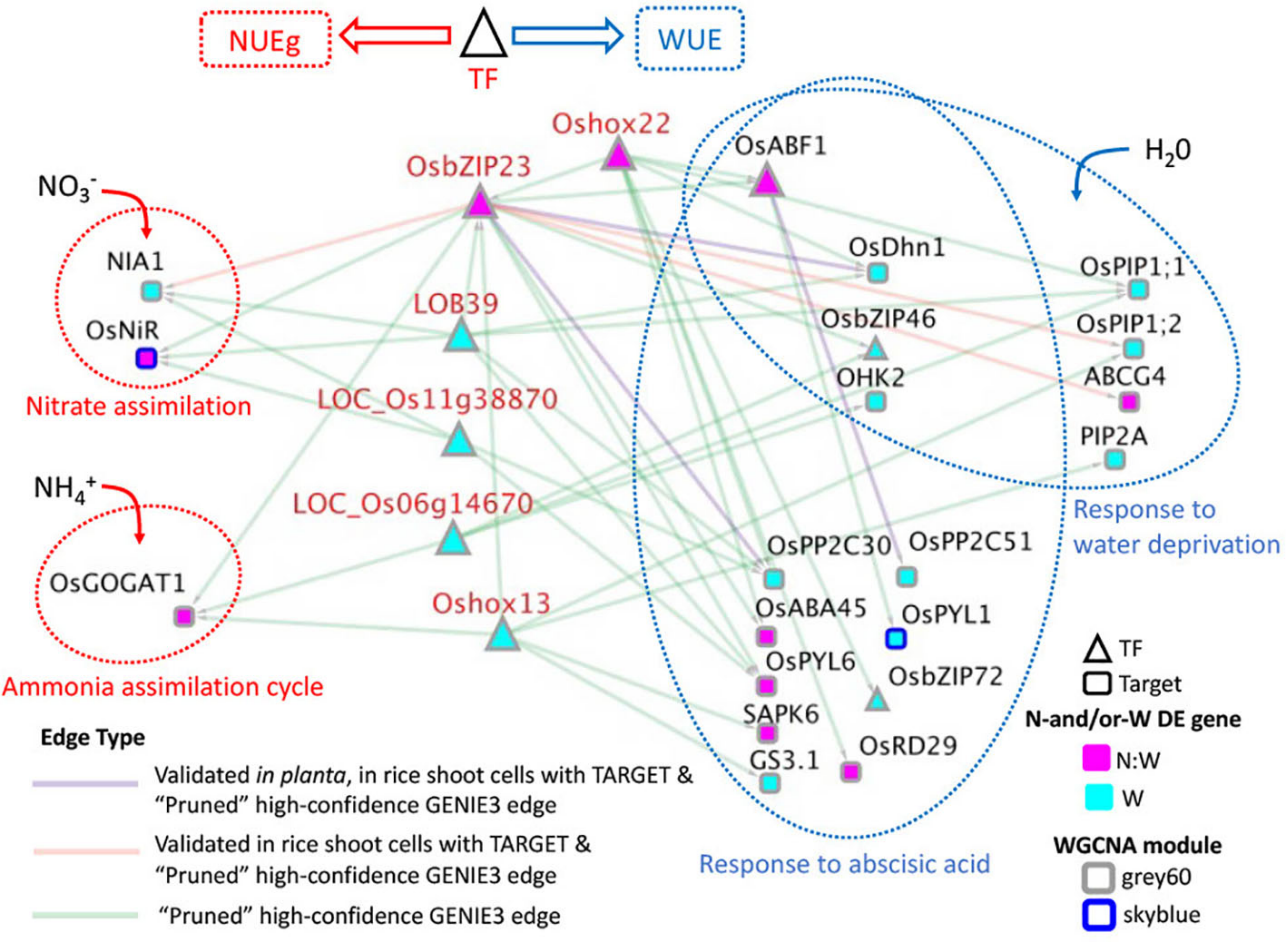
High-confidence GRN of rice TFs Targeting N-and/or W response DE genes correlated with NUEg connected to nitrogen and drought GO terms. This network consists of the TFs from **Table 1** that regulate target genes associated with the gene ontology (GO) terms, "nitrate assimilation" (GO:0042128), "ammonia assimilation cycle" (GO:0019676), "response to water deprivation" (GO:0009414), and "response to abscisic acid" (GO:0009737). These GO terms were selected based upon the enrichment of these terms in the TF-target genes for each TF candidate from **Table 1** using g:Profiler (Raudvere et al., 2019). The full list of GO terms for each TF is in **Supplementary Data 8**. To create this network the 551 total target genes from **Table 1** were examined for the genes associated with the selected GO terms. This left 23/551 target genes and 14/18 TFs from **Table 1** that regulate them. For simplicity and significance, we highlight the 6 TFs in red and their target genes because they regulate genes related to both nitrogen and water, either directly or indirectly. All 6 TFs were also associated highly with NUEg and WUE (**Table 1**). Edges for this network include either high-confidence GENIE3 edges, or validated GENIE3 edges for OsbZIP23 and OsABF1 for which we had TARGET data, and *in planta* data. The total network is in list in **Supplemental Data 7**.

The GENIE3 package in R (Huynh-Thu et al., 2010) was used for network inference analysis. The gene expression data used to make the GENIE3 network consisted of the normalized counts of 228 RNA-seq samples for 10,815 N-and/or-W DE genes from Swift et al., 2019 (Swift et al., 2019) (**Figure 1** Step 3). The 90 TFs for GENIE3 were selected from the two WGCNA modules (grey60 and skyblue) that are highly correlated with NUEg and are also N-and/or-W responsive (**Figure 3D** and **Supplementary Data 4**). The total unpruned network of 973,260 edges were uploaded to ConnecTF-Rice (rice.connectf.org) for network pruning and AUPR analysis (Brooks et al., 2020). This analysis is based on the *in planta* TF-target gene validation data for OsbZIP23, OsABF1, and OsNAC14 that is housed in the ConnecTF database (Brooks et al., 2020) (**Figure 4** and **Supplementary Figure 2**). Gene Ontology (GO) biological process analysis was conducted using g:Profiler (https://biit.cs.ut.ee/gprofiler/gost) with settings for only annotated genes and a significance threshold of 0.05 calculated with Benjamini-Hochberg FDR (Raudvere et al., 2019) (**Table 1**). For this analysis the gene IDs for target genes and genes associated with GO terms were converted between MSU7 and RAPDB gene designations. Cytoscape v3.9.1 was used for network visualization (Paul Shannon et al., 1971) (**Figure 5**).

**Table 1:** Ranked list of 18 prioritized TFs that correlate with NUEg based on high-confidence edges to N-and/or-W DE genes in WGCNA modules (grey60 and skyblue).

Rank. TF Name	Significant overlap of pruned GENIE3 target genes w/1,099 N-and/or-W DE genes in WGCNA modules(grey60&skyblue) #TF_2_s / #genes (Z score)	Relevant N and/or W GO terms associated with TF-target genes that overlap with N-and/or-W DE genes in WGCNA modules (grey60&skyblue)	TFs with High GS and MM for NUEg &/or WUE in WGCNA	Published TF Function (Reference)
1. OsbZIP23	17 TF_2_s/159 genes (52.2)	"Response to water deprivation"	NUEg & WUE	Drought tolerance (Xiang 2008; Dey 2016; Zong 2016)
2. Oshox22	11 TF_2_s/93 genes (39.3)	"Response to water deprivation" & "Response to abscisic acid"	NUEg & WUE	Drought tolerance (Zhang et al., 2012)
3. LOB39	5 TF_2_s/53 genes (30.9)	"Nitrate assimilation"	NUEg & WUE	N-responsive gene (Obertello 2015; Yang 2017)
4. Oshox13	5 TF_2_s/52 genes (27.6)	"Response to water deprivation"	NUEg & WUE	Unknown/Novel
5. LOC_Os11g38870	0 TF_2_s/37 genes (25.9)	"Nitrate assimilation"	NUEg & WUE	Unknown/Novel
6. LOC_Os06g14670	4 TF_2_s/49 genes (24.2)	"Response to water deprivation" & "Ammonia assimilation cycle"	NUEg & WUE	Unknown/Novel
7. ERF65	7 TF_2_s/53 genes (32.1)	No N and/or W GO terms found	NUEg & WUE	Unknown/Novel
8. OsERF48	6 TF_2_s/57 genes (27.3)	No N and/or W GO terms found	NUEg & WUE	Drought tolerance (Jung 2017)
9. OsIRO3	2 TF_2_s/24 genes (16.4)	No N and/or W GO terms found	NUEg & WUE	Iron homeostasis (Wang 2020)
10. LOC_Os03g08470	1 TF_2_/20 gene (15.2)	No N and/or W GO terms found	NUEg & WUE	Unknown
11. OsERF1	4 TF_2_s/25 genes (15.2)	No N and/or W GO terms found	NUEg & WUE	Ethylene response (Hu 2008)
12. OsABF1	5 TF_2_s/61 genes (13.6)	No N and/or W GO terms found	NUEg & WUE	Drought tolerance (Zhang 2017)
13. OsIRO2	1 TF_2_/15 genes (13.3)	No N and/or W GO terms found	NUEg & WUE	Iron homeostasis/ N-signaling (Ogo 2007; Ueda 2020)
14. OSBZ8	1 TF_2_/19 genes (12.8)	No N and/or W GO terms found	NUEg & WUE	ABA response (RoyChoudhury 2008)
15. RSR1	4 TF_2_s/18 genes (10.2)	No N and/or W GO terms found	NUEg & WUE	Starch biosynthesis (Fu 2010)
16. OsSPL9	0 TF_2_s/15 genes (10.0)	No N and/or W GO terms found	NUEg & WUE	Grain yield (Hu 2021)
17. EIL4	4 TF_2_s/40 genes (18.4)	No N and/or W GO terms found	NUEg	Unknown/Novel
18. IDEF2	5 TF_2_s/96 genes (10.7)	No N and/or W GO terms found	NUEg	Iron homeostasis (Ogo 2008)
	**Total** 52 TF_2_s/551 genes	"Response to water deprivation" & "Response to abscisic acid"		

First the TFs were ranked by the Z score for the overlap between the TF→target genes and the 1,099 N and/or W DE genes in WGCNA modules (grey60 & skyblue) associated with NUEg; second, for if the overlapping target genes for each TF were enriched for nitrogen and/or water GO terms; and third, for if the TF was highly associated with both NUEg and WUE.

### Plasmid construction for TF-perturbation experiments using *TARGET* assay in plant cells

The coding sequences of OsABF1 and OsbZIP23 were determined as listed in Phytozome 13 (Goodstein et al., 2012) and were synthesized by GENEWIZ (South Plainfield, NJ) with the GATEWAY cassette for cloning into the p1107 destination plasmid (**Supplementary Figure 4**). Entry vectors were cloned into the p1107 plasmid using the LR Clonase II reaction according to manufacturer's instructions (Invitrogen). The p1107 plasmid for rice TARGET has a pBeaconRFP_GR (Bargmann et al., 2013) backbone with the following modification. The 35S promoters were replaced with maize Ubiquitin promoter subcloned from pTDM-C (Wu et al., 2016). A biotin ligase recognition peptide (BLRP) was fused at the N-terminal of the GATEWAY cassette, which is followed by the GR protein. All junctions were sequenced and verified for in frame TF-GR fusion proteins. The plasmid map and sequence (.FASTA) are provided in **Supplemental Data File 1**.

### TARGET temporal TF perturbation experiment in rice leaf cells and RNA-sequencing

The rice protocol was adapted from our Arabidopsis *TARGET* protocol (Bargmann et al., 2013; Brooks et al., 2019) with some modifications. Rice seeds (Nipponbare) were sterilized by 70% ethanol for 3 mins followed by 50% commercial bleach for 30 min with rotation. The rice seeds were germinated in the dark for 4 days. The germinated rice seeds were transferred to ½ MS plates without sugar for 13 days in the growth chamber, under 16 h light/8 h dark diurnal cycle, at temperatures 27 and 25°C respectively and 70% humidity. On the day of the TARGET experiment, rice shoot tissue was cut into small (1 mm) pieces and stirred with cell-wall digestion solution (1.5% cellulase RS, 0.3% macerozyme R10 (Yakult Honsha), 0.6M mannitol, 10 mM MES (pH 5.7), 1 mM CaCl_2_, 5 mM b-mercaptoethanol, and 0.1% BSA) in a flask. The flask was vacuumed infiltrated for 20 minutes and shaken at 50 rpm in the dark for 4 hours. Rice shoot protoplasts were filtered through a 40 µm cell strainer (BD Falcon, USA) and spun down for 5 min at 500 g. The rice shoot protoplasts were then washed with 10 mL W5 solution (150 mM NaCl, 1M CaCl_2_, 1M KCl, 200 mM MES pH 5.7) three times, then resuspended in MMG solution (400 mM D-mannitol, 10 mM MgCl_2_, 4 mM MES pH 5.7) to 1.0x10^6^ cells/mL. For protoplast transfection with vector, 1.0x10^5^ cells were mixed with 40 µg plasmid DNA and 110 µL 40% PEG solution (40% 4000 PEG (Sigma, 81242), 400 mM D-mannitol, 50 mM CaCl_2_). The mixture was incubated at room temperature for 10 minutes. After incubation, the protoplasts were washed with W5 solution three times and resuspended in 1 mL WI solution (400 mM D-mannitol, 1M KCl, 200 mM MES pH 5.7). The transfected protoplasts were stored in the dark overnight. The next day, transfected protoplasts were treated with 30 µM cycloheximide (CHX) for 20 minutes (to block translation of secondary TF_2_ targets genes), before a three-hour 10 mM dexamethasone (DEX) treatment (to induce TF-GR nuclear import). After 3 hours, TF vector and control empty vector transfected protoplasts were FACS sorted for RFP signals into 150 µL TRI regent for RNA extraction (Zymo, R2061) (**Supplementary Figure 5**). We used Lexogen QuantSeq 3' mRNA-Seq Library Prep Kit FWD for Illumina (Lexogen, 015.2x96) for making RNA-Seq libraries. The libraries were pooled and sequenced on the Illumina NextSeq 500 platform at NYU-CGSB Genomics Core facility.

### RNA-seq analysis of TARGET assay for validation of TF-target direct regulated genes

The UMI-incorporated RNA-Seq libraries of TF-transfected and empty vector control were analyzed following Lexogen's guidance (https://www.lexogen.com/quantseq-3mrna-sequencing/). The reads' UMI were extracted from raw fastq files using `*extract*` command from UMI-tools v1.1.1 (Smith et al., 2017). Then the fastq files were trimmed by fastp 0.21.0 (Chen et al., 2018). The clean fastq files were aligned to MSU7 (Kawahara et al., 2013) genome using STAR 2.7.6a (Dobin et al., 2013). The aligned reads with the identical UMI were deduplicated using `*dedup*` command from UMI-tools v1.1.1 (Smith et al., 2017). The gene counts matrix was generated by featureCounts v2.0.1 (Liao et al., 2014) from the deduplicated bam files. The TARGET DE genes for OsABF1 and OsbZIP23 were identified using DESeq2 package (Love et al., 2014) by comparing TF vs Empty Vector with a Benjamin & Hochberg adjusted p-values< 0.05. Differentially expressed (DE) genes identified for OsABF1 and OsbZIP23 are listed in **Supplementary Data 9**. Overlap between *in planta* and TARGET data was conducted with Venny 2.1 (Oliveros, 2015) and the significance was determined with Genesect in Virtual Plant 1.3 (Katari et al., 2010). The calculations for precisions, recall and F-score for the GENIE3 network was the same as in Brooks et al., 2019 (Brooks et al., 2019) (**Supplementary Figure 6**).

## Results

### Phenotypic variation in NUEg in 19 rice varieties grown in N-by-W matrix field

The N-by-W response field data set used in our current study consisted of 19 rice varieties treated in a 2x2 matrix of four N-and/or-W treatment conditions (**Figure 1**) (Swift et al., 2019), comprising: well-watered (HW) with low-or-high N (HWLN, HWHN) (**Figure 2A**) vs. Low-W (LW) with low-or-high N (LWHN, LWLN) (**Figure 2B**) (For treatment details see Materials & Methods, and Swift et al., 2019. To refine our focus to NUEg, we examined how each of the 19 rice varieties performed for NUEg in the field (**Figure 2**). To identify the rice varieties with higher NUEg in the four different N-by-W field conditions, we adapted the Potential Index (I_PO_) (Ndiaye et al., 2019) of NUEg for our N-by-W field dataset (**Figure 2**). The I_PO_ for NUEg indicated the relative performance of each of the 19 rice varieties, compared to the conditional average (dotted lines). Under the well-watered (HW) condition, none of the rice varieties performed well under *both* LN *and* HN conditions (**Figure 2A**). For example, IR64 showed the highest NUEg values under HWLN, but only average NUEg values under HWHN conditions (**Figure 2A**). By contrast, Tainung67 showed the highest NUEg values under HWHN, but only average NUEg values under HWLN conditions (**Figure 2A**). However, under LW treatments, there was one variety, IR108, that performed well under *both* LWLN *and* LWHN conditions, with the highest I_PO_-NUEg compared to the other 18 varieties (**Figure 2B**). In line with this finding, the IR108 variety has been released under the variety name "Sukha dhan 5" to be used in the drought-prone regions of Nepal (Anantha et al., 2016). The I_PO_ analysis reveals that this phenotypic dataset covers a range of rice NUEg values. Therefore, we used this NUEg phenotype data from the 2016 growing season data and the corresponding transcriptome data of Swift et al 2019, for the ensuing network-to-NUE phenotype analysis (**Figure 1**).

### Identification of N-and/or-W responsive DE genes highly correlated with NUEg

To discover the relationships between genes and field phenotypes including NUEg, we used WGCNA (Langfelder and Horvath, 2008) (**Figure 1**, Step 2, and **Figure 3**). The WGCNA analysis identified 11 co-expression modules for the 22,436 genes from the rice transcriptome data from the N-by-W field plot (**Figure 1**, Step 2, **Figure 3A**, Data in **Supplemental Data 1**). The genes in each of the WGCNA co-expression modules contribute to a Module Eigengene (ME) value based upon their Module Membership (MM) score. The MM score is the contribution of the individual gene to the ME value of the module (Langfelder and Horvath, 2008). We used the ME value to determine module correlation with each of the rice phenotypes from the N-by-W field plots (**Figure 3A**). The ME score for two WGCNA modules, grey60 (3,050 genes) and skyblue (744 genes) was significantly and highly correlated with the NUEg and WUE phenotype data in the N-by-W plot (**Figure 3A**). The ME value of the grey60 module was negatively correlated with NUEg (-0.71), while the ME value of the skyblue module was positively correlated with NUEg (+0.73) (**Figure 3A**). However, each WGCNA module contains subsets of genes that can be either positively or negatively correlated with NUEg. In WGCNA, this gene expression-to-phenotype correlation is called Gene Significance (GS), as shown for the plot of MM vs. GS in **Figure 3C**.

To identify which WGCNA modules had a significant representation of genes responding to N-and/or-W signals, the genes comprising each module were overlapped with the N-and/or-W responsive DE genes from Swift et al 2019 (Swift et al., 2019) (**Figure 3B**). This analysis uncovered a significant overlap of the N:W- and W- responsive gene lists with the genes in the WGCNA modules - grey60 and skyblue - which are each highly correlated with NUEg and WUE (**Figure 3A**). This demonstrates that the genes in the WGCNA modules - grey60 and skyblue - not only correlate with the NUEg phenotypes from the N-by-W matrix field plots but are *also* enriched in genes responsive to N-and/or-W signals (**Figure 3B**). Additionally, the blue and lightyellow WGCNA modules are enriched in genes that respond to N-moles, but not to the interaction of N and W. While the WGCNA modules - blue and lightyellow - do not correlate significantly with NUEg, each of these modules correlates significantly with chlorophyll concentration (**Figure 3A**), a trait known to be regulated by N and used to determine N-status and the need for fertilizer in the field (Fageria et al., 2010).

Next, we performed two analyses that enabled us to prioritize the N-and/or-W response DE TFs and genes within each of the two WGCNA modules - grey60 and skyblue - that are most highly correlated with the NUEg phenotype (**Figures 3C, D**). The genes with MM scores closes to -1 or 1 are highly connected to their WGCNA module. In addition, genes with GS scores that have a high absolute value for a specific trait are also more biologically significant (Langfelder and Horvath, 2008). Therefore, to filter genes in each module that were highly correlated with NUEg, we identified genes with high absolute values for both their MM and GS scores. To do this, we first plotted the absolute values of the MM vs. GS scores for each gene in the WGCNA modules - grey60 and skyblue - which are highly correlated with NUEg (**Figure 3C**). Next, we calculated the average MM and GS scores for the genes in each of these two modules. This enabled us to set a cut-off and identify genes whose absolute MM and GS values were great than or equal to the average of the genes in each module (**Figure 3C**, upper right quadrant).

This analysis identified a combined total of 131 TFs and 1,491 genes highly relevant to NUEg in the two WGCNA modules: grey60 (104 TFs & 1,209 genes) and skyblue (27 TFs & 282 genes) (**Figure 3C**). Next, to identify whether genes highly relevant to NUEg are significantly enriched in N-and/or-W response gene, we performed a Genesect analysis (Katari et al., 2010) (**Figure 3D**). This analysis revealed significant overlaps between the N:W and W responsive gene lists from Swift et al 2019 (Swift et al., 2019), with the genes highly correlated with NUEg (131 TFs and 1,491 genes) from the combined grey60 and skyblue WGCNA modules (**Figure 3D**). The resulting overlap consisted of 90 TFs and 1,099 genes that are highly associated with NUEg and N-and/or-W responsive (**Supplementary Data 2**). Next, we determined which of these TFs and genes correlated NUEg were also highly associated with the WUE phenotype. To do this, we conducted the same analysis pipeline as described above for NUEg, in which we determine a new GS cut off value for WUE (**Supplementary Figure 1A**). This resulted in 79 TFs and 976 genes that are highly correlated with WUE and are N-and/or-W responsive (**Supplementary Figure 1B**, **Supplementary Data 3**). We find that 72 (80%) NUEg TFs and 815 (74%) NUEg genes are also highly correlated with WUE, thus suggesting a dual role for these genes/TFs in regulating both N and W responses.

For further analysis, we prioritized 90 TFs from the GENIE3 analysis that are; i) N-and/or-W responsive and ii) highly correlated to NUEg from the combined WGCNA modules - grey60 and skyblue. This analysis resulted in 29 TFs that are N:W-responsive and 61 TFs that are W-responsive (**Figures 3C, D**).

### Validation of TF→target GRN predictions in WGCNA modules associated with NUEg

To predict TF→target gene interactions in GRNs important for NUEg, we used GENIE3, a random forest network inference method (Huynh-Thu et al., 2010). This analysis will identify potential master TF regulators of the NUEg response amongst the 90 TFs (29 TFs N:W-responsive and 61 TFs W-responsive) (**Figure 3D**) that are highly correlated with NUEg (e.g., members of WGCNA grey60 and skyblue models) (**Figures 3A**, **C**). To identify and rank these 90 TFs from these NUEg modules, we generated a GRN using 90 potential TF-regulators of 10,815 DE (N-and/or-W response genes) from the field N-by-W matrix (**Figure 1**, Step 1). The output of GENIE3 ranks the TF→target gene predictions in the order of confidence for each of the 90 TFs and the 10,815 DE genes N-and/or-W responsive (**Figure 4**). In total, the resulting GENIE3 inferred network ranks numerical confidence scores for each TF and target gene, excluding self-regulation of the TF ((90 TF x 10,815 genes) - 90 TFs) = 973,260 TF-target edges (**Figure 4** and **Supplemental Data 4**).

Our next goal was to validate the TF-target gene interactions in our predicted GRN, using TF-target gene data validated *in planta*. To this end, we used experimentally validated TF-target gene interactions from TF perturbation data in rice, housed in the ConnecTF platform (https://rice.connectf.org) (Brooks et al., 2020) (**Figure 4** and **Supplementary Figure 2**). The ConnecTF database includes published rice RNA-seq and ChIP-seq data available as of June 2020. To validate the GRN, we uploaded the TF→target gene interactions predicted by the GENIE3 network into ConnecTF and filtered for validated TF-regulation (RNA-seq) and TF-binding (ChIP-seq) data from rice *in planta* datasets (**Figure 4** and **Supplementary Figure 2**, **Supplementary Data 4** **)**. We focused our analysis on validated TF-target gene datasets from rice leaf tissue, given that the source RNA-seq data used to make the GENIE3 network was from rice leaves (**Supplementary Figure 2**).

Our query of the ConnecTF rice TF database identified experimental TF-target gene validation datasets for three TFs in rice leaf tissue from our GENIE3 network (**Figure 4** and **Supplemental Figure 2** **).** The three validated rice TFs are OsABF1 (Zhang et al., 2017), OsbZIP23 (Zong et al., 2016), and OsNAC14 (Shim et al., 2018). These three validated rice TFs include a total of nine datasets with 10,941 validated target genes from TF-regulation and/or TF-binding data (**Figure 4** and **Supplementary Figure 2**). We then used this *in planta* data as "gold-standard" data to validate the TF→target gene predictions from our GENIE3 network using Area Under the Precision Recall (AUPR) curve analysis, which is an automated function in the ConnecTF platform (**Figure 4**). The results show that the AUPR for the TF→target gene predictions (edges) in the rice GENIE3 network were significantly higher than the random TF-target gene edges (P-value<0.001, permutation test) (**Figure 4**). Given the AUPR curve, we were able to select a precision threshold of 0.31 (e.g., TF→target gene edge score ≥ 0.0581). This cut-off score is equivalent to the TF→target gene predictions being accurate 1/3 of the time and this level of accuracy is comparable to other similar network validation AUPR studies (Varala et al., 2018; Brooks et al., 2019). The GENIE3 network was then pruned for only the high-confidence TF→target gene predictions using this precision cut-off score. This network pruning for precision, resulted in a GRN containing 8,826 high confidence edges connecting 88 TFs and 5,716 target N-and/or-W response DE genes (**Figure 4** and **Supplementary Data 5**).

### Prioritization of master TFs that regulate NUEg in response to N-and/or-W signaling

Our next goal was to prioritize candidate N-and/or-W response TFs with a significant influence on NUEg from the pruned GENIE3 network. To this end, we overlapped the pruned high confidence TF→target edges for the 88 TFs in the GENIE3 network with the 1,099 genes from the two WGCNA modules that are highly correlated with NUEg - grey60 & skyblue - N-and/or-W DE genes = 322 N:W response genes + 777 W-response genes) (**Supplementary Figure 3**). We calculated the significance of the overlapping TF→target genes with the 1,099 NUEg genes. To prioritize the 88 TFs, we ranked them by the Z-score for the overlap (**Supplementary Data 6**). We found 18 TFs whose high confidence TF→targets gene edges had the highest significant overlap (P-value<0.001, Z score ≥ 10) with the 1,099 genes in the NUEg WGCNA modules – grey60 and skyblue (**Table 1**). This analysis links 18 TFs→ 551 N-and/or-W response target genes→NUEg. Among the 18 TFs, OsbZIP23 is predicted to regulate the most of the NUEg correlated genes, compared to the other 17 TFs (**Table 1**). Additionally, we find that 16/18 TFs (all except EIL4 and IDEF4) are also highly corelated with WUE (**Table 1** and **Supplemental Data 3**).

Of these 18 TFs, multiple TFs have published functions in drought tolerance including, OsABF1 (Zhang et al., 2017), OsbZIP23 (Xiang et al., 2008; Dey et al., 2016; Zong et al., 2016), Oshox22 (Zhang et al., 2012), and OsERF48 (Jung et al., 2017). Of note, OsABF1, OsbZIP23, and Oshox22 are N:W-responsive genes based on the N-and/or-W response DE gene lists from Swift et al 2019 (**Supplemental Data 6**), suggesting their new function in regulating N:W responses, in addition to drought (**Table 1**). Published functions for other candidate TFs in the 18 TF list include, N-signaling (LOB39) (Obertello et al., 2015; Yang et al., 2017), ABA signaling (OSBZ8) (RoyChoudhury et al., 2008), ethylene signaling (OsERF1) (Hu et al., 2008), iron homeostasis (IDEF2, OsIRO3, and OsIRO2) (Ogo et al., 2007, 2008; Masuda et al., 2019; Wang et al., 2020), starch biosynthesis (RSR1) (Fu and Xue, 2010), and grain yield (OsSPL9) (Hu et al., 2021) (**Table 1**). OsIRO2 was also found to regulate NUE in a N-response gene network in rice (Ueda et al., 2020).

### Gene ontology for target genes for prioritized TFs

To further determine the mechanism of the prioritized TFs in regulating NUEg, we performed Gene Ontology (GO) analysis on the NUEg target genes from **Table 1** regulated by each TF using g:Profiler (**Table 1** and **Supplemental Data 7**) (Raudvere et al., 2019). For each TF, we focused on the relevant biological process GO terms related to water and nitrogen signaling. We found that the target genes of the TFs, LOB39, LOC_Os11g38870, and LOC_Os06g14670, were enriched for GO terms related to nitrogen including, "nitrate assimilation," and "ammonia assimilation cycle" (**Table 1**). Further, we found that the target genes of the TFs, OsbZIP23, Oshox22, Oshox13, LOC_Os06g14670, were enriched for GO terms related to drought including, "response to water deprivation," and "response to abscisic acid" (**Table 1**). LOC_Os06g14670 was the only TF enriched for nitrogen and drought-related GO terms. We did not identify any GO enrichment for the TF-target genes of OsERF48, OsIRO3, LOC_Os03g08470, OSBZ8, RSR1 and IDEF2. However, we did identify some other GO terms of interest for the remaining TFs including, "sulfur compound metabolic process" for EIL4, "cell communication" ERF65, "response to temperature stimulus" for OsABF1, "phosphorus metabolic process" for OsERF1, "iron ion homeostasis" for OsIRO2, and "zinc ion homeostasis" for OsSPL9 (**Supplemental Data 8**). While these enriched GO terms suggest the relevance of these TFs in other cell processes, we focus on the TFs that regulate the target genes associated with the nitrogen and water related GO terms.

### High-confidence GRN of TFs that target nitrogen and drought-related genes

To identify the TFs that regulate both nitrogen and water response from our list of prioritized TFs, we took the subset of the GENIE3 network that includes 18 TFs→ 551 N-and/or-W response target genes associated with NUEg, and identified the target genes from this list of 551 that were part of the GO terms "nitrate assimilation", "ammonia assimilation cycle", "response to water deprivation," and "response to abscisic acid" (**Supplemental Data 7**). This resulted in a list of 23 target genes regulated by 14 TFs (**Supplemental Data 7** **)**. We found six TFs that regulated both nitrogen and water related target genes either directly (OsbZIP23, LOB39, LOC_Os11g38870, LOC_Os06g14670, and Oshox13) or indirectly (Oshox22 via regulation of OsbZIP23) (**Figure 5**). While OsABF1 did not regulate genes related to nitrogen, it is included in the network visualization because it is annotated for the water-related GO terms and is regulated by OsbZIP23 and Oshox22 (**Figure 5**).

The target genes involved in nitrate and ammonia assimilation that are regulated by the TFs in our high-confidence GRN include validated regulators of NUE, glutamate synthetase 1 (OsGOGAT), and nitrite reductase (OsNiR) (Lee et al., 2020; Yu et al., 2021) (**Figure 5**). We also find regulation of the putatively expressed nitrate reductase 1 (NIA1) gene, which is necessary for nitrate assimilation (Subudhi et al., 2020). The TFs, OsbZIP23, LOB39 and LOC_Os11g38870 regulate nitrate assimilation genes, while OsbZIP23, Oshox13, and LOC_Os06g14670 regulate the ammonia assimilation gene. OsbZIP23 is the only TF that regulates genes in both nitrate and ammonia assimilation genes (**Figure 5**).

Furthermore, each TF regulates genes involved in water deprivation and/or ABA signaling (**Figure 5**). These genes include the TFs OsbZIP46 and OsbZIP72, which are known positive regulators of drought tolerance and function in coordination with OsbZIP23 and OsABF1, two other prioritized TFs in our network (Lu et al., 2009; Chang et al., 2017; Zhang et al., 2017; Song et al., 2020). We also find regulation of the rice aquaporins, OsPIP1;1, OsPIP1;2, and PIP2A that facilitate water transport (Sakurai et al., 2005; Xu et al., 2019). In addition, there are genes that regulate multiple components involved in the ABA signaling pathway including, the ABA drought receptors, OsPYL1, OsPYL6 (Li et al., 2015; Santosh Kumar et al., 2021a), the clade A type 2C protein phosphatases, OsPP2C51, OsPP2C30 (Zong et al., 2016; Santosh Kumar et al., 2021a), and the ABA-activated protein kinase, SAPK6 (Chang et al., 2017). Overall, this result demonstrates that a subset of our prioritized candidate TFs regulates both nitrogen and water genes.

### Network validation with *in vivo* TARGET assay

To further validate the nitrogen and drought-related edges in our high-confidence GRN (**Figure 5**), we performed *in vivo* Transient Assay Reporting Genome-wide Effects of Transcription factors (*TARGET*) assays to identify the *direct* TF-target genes for these TFs. We selected OsbZIP23 and OsABF1 for TARGET assays because we could compare the accuracy of our TARGET results with the available *in planta* data for these TFs in ConnecTF (Brooks et al., 2020). The *TARGET* TF-perturbation assay in isolated plant cells has been previously used to identify direct TF→regulated target genes in Arabidopsis (Bargmann et al., 2013; Varala et al., 2018; Brooks et al., 2019). In this paper, we adapt the vectors and the *TARGET* temporal TF-perturbation assay to rice shoot cells (**Supplementary Figure 4**).

The *TARGET* TF-perturbation assay identifies the direct TF→ regulated target gene interactions because; i) there is timed nuclear entry of the TF, and ii) translation of regulated secondary (TF_2_) transcription factors is blocked by cycloheximide treatment. TF-regulated DE genes are identified by comparison to an empty vector control. The *TARGET* assay identifies direct TF→target genes as follows: i) the TF is fused to the glucocorticoid receptor (GR) protein that when expressed in the plant cells, ii) the TF-GR fusion is retained in the cytoplasm by HSP90 binding, iii) upon dexamethasone (DEX) treatment, the GR binding is released and the TF is imported into the nucleus where it can regulate expression (Bargmann et al., 2013; Brooks et al., 2019) (**Supplementary Figure 5**). iv) Additionally, cycloheximide + DEX treatment inhibits translation of mRNA for TF_2_s. Therefore only the target genes of the over-expressed TF are identified, when compared to the empty vector control (Brooks et al., 2019).

Based on our TARGET assay, OsbZIP23 directly regulates 3,095 target genes, while OsABF1 directly regulates 2,151 target genes in rice shoot protoplasts (**Supplementary Figure 6** and **Supplemental Data 9**). To determine the accuracy of our TARGET results, we took the overlap between the TARGET results and the *in planta* binding and expression data for each TF from ConnecTF (Zong et al., 2016; Zhang et al., 2017; Brooks et al., 2020). We found a significant overlap between the TARGET and *in planta* DE genes (**Supplemental Figure 6A**). This significant overlap suggests that the plant cell-based TF-target data can accurately identify *in planta* TF-regulated genes. Additionally, we find the *TARGET* data is as accurate, if not even better, than the *in planta* data at validating the predicted TF→target genes in the GENIE3 network, with a higher F-score and similar precision and recall values (**Supplementary Figure 6B**).

Given that the TARGET data was accurate in identifying OsbZIP23 and OsABF1 target genes, we used the TARGET and *in planta* data to validate the nitrogen and drought-related edges in our high-confidence GRN (**Figure 5**). We confirm with TARGET that OsbZIP23 directly regulates genes involved in nitrogen and drought responses including, NIA1 involved in nitrate assimilation (Subudhi et al., 2020), ABCG4 involved in abiotic stress responses (Matsuda et al., 2012), and the rice aquaporin, OsPIP1;2, that improves yield in rice (Xu et al., 2019). Additionally, we confirm with OsbZIP23 TARGET and *in planta* data that OsbZIP23 regulates drought associated genes OsDhn1 and OsPP2C30 (Lee et al., 2013; Santosh Kumar et al., 2021b). Furthermore, we confirm the role of OsABF1 in regulating drought signaling, as it regulates the drought-associated gene OsPP2C51 in both TARGET and in planta datasets (**Figure 5**) (Zong et al., 2016).

Overall, our TARGET results show that the high-confidence edges inferred in our GENIE3 network accurately predict TF→target genes, thus further confirming the role of OsbZIP23 in regulating both NUEg and WUE. In addition, we find a new function for OsbZIP23 in mediating NUEg phenotypes, as previous studies show its role in drought responses (Xiang et al., 2008; Dey et al., 2016; Zong et al., 2016). Thus, our combined network inference and validation approach reveals new TFs in regulating NUEg (**Table 1**).

## Discussion

In this study, we sought to identify GRNs that control NUEg in response to two key interacting components that regulate rice productivity: N and W. By exploiting transcriptomic and phenotypic data collected from 19 rice varieties grown in a 2x2 N-by-W matrix in the field (Swift et al., 2019), we identified and validated the role GRNs comprised of N-and/or-W response genes for their role in TF→target gene→ NUEg phenotype relationships. The TF to N-by-W response gene information now encoded in this high-confidence GRN correlated to NUEg, can now be applied to develop/breed rice plants with improved yield marginal, low N-input, drought-prone soils – which are increasing in the face of climate change.

### High-confidence GRN identifying master regulators of NUEg responsive toN-and/or-W signals

We were able to link the TF→target gene→NUEg phenotype using a combination of four approaches (*i*) WGCNA-based gene-to-trait co-expression network (Langfelder and Horvath, 2008), (*ii*) GENIE3, a random forest machine learning approach to GRN inference for predicting TF-target interactions (Huynh-Thu et al., 2010), (iii) Experimental validation of GRN predictions and Network "pruning" by AUPR (Varala et al., 2018; Brooks et al., 2019), and (iv) Network validation using TARGET, an approach which uses plant cells to identify *direct* TF→target gene interactions (Bargmann et al., 2013; Brooks et al., 2019). Using this pipeline (**Figure 1**), the WGCNA approach identified two network modules that were highly correlated to NUEg called "grey" and "skyblue". Next, we constructed a GRN for the genes in this module, based on their N-and/or-W response DE genes. Finally, we used experimental data for TF-target genes validated *in planta* (Zong et al., 2016; Zhang et al., 2017; Shim et al., 2018) as well as ones we generated in rice leaf cells for this study. These validated rice TF datasets were used to conduct precision/recall analysis of our GRN. This enabled us to set a precision cut-off score to prune the network for high confidence TF-target predictions for *all* TFs in the GRN.

Overall, our GRN analysis and validation identified OsbZIP23 and Oshox22 as top candidate master regulators of NUEg in response to N and W signaling. These two TFs are network hubs, as they regulate the largest number of DE genes (N-and/or-W responsive) that are highly correlated with NUEg in the grey60 and skyblue WGCNA modules (**Table 1** and **Supplemental Data 6**). Further validating their known role in drought, these two TFs have published functions in regulating drought tolerance through the plant hormone abscisic acid (ABA) signaling responses (Xiang et al., 2008; Zhang et al., 2012, 2017; Park et al., 2015) (**Table 1**). Our current study, now links these two well-known drought TFs to regulation by N-and/or-W signaling and NUEg. Our results are also in line with previous studies that show OsbZIP23 activity to be dependent upon phosphorylation by SAPK2 (Zong et al., 2016), an osmotic stress/ABA-activated protein kinase, which promotes nitrate uptake and assimilation under drought stress (Lou et al., 2020).

In addition to the TF hubs (OsbZIP23 and Oshox22), we identify four TFs with novel functions NUEg and WUE gene regulation in our GRN. We identified four TFs (LOB39, Oshox13, LOC_Os11g38870, and LOC_Os06g14670), that regulate genes involved in both N and/or W responses using GO analysis of their predicted TARGET genes in the high-confidence GRN (**Table 1** and **Figure 5**). Unlike OsbZIP23 and Oshox22, the TFs Oshox13, LOC_Os11g38870, and LOC_Os06g14670TFs had until now unknown functions in both nitrogen and drought regulation (**Table 1**). LOB39 expression is regulated by nitrogen, however it was previously not known to be involved in drought (Obertello et al., 2015). OsbZIP23, LOB39 and LOC_Os11g38870 regulate nitrate assimilation genes NIA1 and OsNiR, which is a known to promote nitrogen assimilation and NUE in coordination with OsNLP4 (**Figure 5**) (Yu et al., 2021). Furthermore, OsbZIP23, Oshox13 and LOC_Os06g14670 regulate the ammonia assimilation gene OsGOGAT1, which improves NUE in low N conditions in coordination with the ammonium transporter OsAMT1;2 (Lee et al., 2020). While it is known that rice prefers ammonia uptake compared to nitrate (Sasakawa and Yamamoto, 1978; Hachiya and Sakakibara, 2017), we find the TFs in this network regulate both pathways, with OsbZIP23 regulating genes involved in both.

We also examined the mechanism of transcriptional regulation between these master TFs in the NUEg GRN by validating TF→target gene interactions using TARGET, a plant cell-based assay that identifies *direct* TF→TARGET gene interactions (Bargmann et al., 2013; Varala et al., 2018; Brooks et al., 2019). We find that Oshox22 regulates nitrogen and water responses indirectly via candidate TFs OsbZIP23, and OsABF1 (**Figure 5**). We then validate the TF→target gene interactions for OsbZIP23 and OsABF1 TFs with the TARGET assay. We confirm that OsbZIP23 regulates both nitrogen and drought response genes, and OsABF1 regulated drought response genes, with TARGET and *in planta* data.

Overall, these finding supports previous studies that show the regulation of these two essential signals N-and-W are linked (Swift et al., 2019; Araus et al., 2020; Plett et al., 2020). Our work presents a path of how ABA/drought induced signaling regulates both N and W responses which ultimately affect crop phenotypes, such as NUEg, the trait of focus in our study.

### Validation of GRNs in rice using ConnecTF as a platform to validate and prune for high-confidence networks

In our study, we demonstrate the usefulness of ConnecTF as a platform - now applied to rice - to integrate published TF-binding and TF-expression datasets to identify and validate target genes in GRNs (Brooks et al., 2020) (**Figure 4** and **Supplementary Figure 2**). While some GRN studies use an arbitrary cut-off value for network pruning as in other network studies (Ueda et al., 2020), we show how TF-perturbation data can be used as a "gold-standard" for GRN validation and "network pruning", using automated AUPR functions in ConnecTF (Brooks et al., 2020) (**Figure 4**). We performed Precision/Recall analysis of the GRN for NUEg – using the TF-target gene validation sets for rice housed in the ConnecTF database. This enabled us to empirically select a TF→target precision cut-off value of 0.31 from the AUPR curve. This AUPR cut-off represents that approximately 1/3 of our GENIE3 network predictions are validated (**Figure 4**). This precision cut-off is comparable to what we find in other network studies in Arabidopsis that use AUPR analysis (Varala et al., 2018; Brooks et al., 2019). Overall, the automated AUPR function in ConnecTF provides an accurate, and facile means to validate GRN predictions in any rice GRN that researchers can load onto the site. Importantly, these cut-off values for TF→target gene validated edges established a cut-off score that can be applied to all TF→target gene edges in the network – including TFs which have not been validated. This enables the generation of a high-confidence network for all TFs in the GRN.

### bZIP family TFs as regulators of N and W signaling

In our high-confidence GRN we identify nine bZIP TFs as regulators in our "pruned" network (**Supplementary Data 6**). Members of the bZIP family of TFs are known to regulate drought stress responses in multiple crops species in addition to rice, including *Glycine max*, *Zea mays* and *Hordeum vulgare* (Joshi et al., 2016). Additionally, bZIP family TFs regulate ABA hormone responses, which play a crucial role in regulating the drought response in plants in general (Joshi et al., 2016; Zong et al., 2016; Zhang et al., 2017; Araus et al., 2020). In our high-confidence GRN studies that focus on genes correlated with NUEg, we find that bZIP TFs regulate N-signaling as well as drought responses in rice. In line with our finding, previous studies examining N-responses in rice, identified bZIP transcription factors that regulate NUE (Ueda et al., 2020).

We identified three bZIP family members - OsABF1, OsbZIP23, and OSBZ8 - as top-regulators of N-and/or-W signaling in regulating NUEg (**Table 1**). Additionally, we find regulation of two other bZIP TFs, OsbZIP72 and OsbZIP46, in our NUEg GRN regulated by Oshox22 and OsbZIP23, respectively (**Figure 5**). This finding is significant, as OsbZIP23, OsbZIP46, OsbZIP72 are part of the same subgroup-III of bZIP TFs and are known to be coordinated in their regulation of ABA signaling and drought responses (Lu et al., 2009; Hossain et al., 2010; Song et al., 2020). Additionally, ObZIP46 improves drought tolerance in coordination with the ABA-activated protein kinase, SAPK6, which is another target gene in our NUEg GRN (**Figure 5**) (Chang et al., 2017). Overall, our NUEg GRN results link bZIP TFs in rice as mediating N-and/or-W response genes that control NUEg. We validate the TF→target genes predictions in our high-confidence GRN for NUEg for two bZIP TFs, OsbZIP23 and OsABF1, using the TARGET assay.

### Functional validation of TFs in rice: TARGET assay to identify direct TF→target gene interactions in rice cells

The TARGET system allows researchers to identify the validated TF-target gene interactions for any TF of interest using a rapid plant cell based temporal TF perturbation assay (Bargmann et al., 2013; Brooks et al., 2019). The key to this assay is the inducible TF nuclear localization and its ability to identify *direct* TF-target genes based on RNA-seq data (Bargmann et al., 2013). Previously, the TARGET assay has been used to identify direct TF→target gene interactions in Arabidopsis root or shoot cells (Bargmann et al., 2013; Varala et al., 2018; Brooks et al., 2019). In this study we establish the TARGET system in rice leaf protoplasts (see Methods). We then used the rice TARGET assay to identify the direct regulated target genes of the rice TFs OsbZIP23 and OsABF1 (**Supplementary Data 9**). Our analysis shows that the TF target genes identified in rice leaf protoplasts using TARGET, are comparable and show a significant overlap with genes identified *in planta* (**Supplementary Figure 6A**). Additionally, in this study, we demonstrate that the accuracy of rice TARGET data is comparable to *in planta* data at validating network predictions (**Supplementary Figure 6B**). This finding suggests that rice TARGET data can be used to validate GRN predictions in rice, as was shown in Arabidopsis (Varala et al., 2018; Brooks et al., 2019; Brooks et al.,2020; Cirrone et al., 2020). In our study, we validated that OsbZIP23 regulates both nitrogen and water-related genes including, NIA1 which is involved in nitrate assimilation (Subudhi et al., 2020), OsDhn1 which is induced by drought (Lee et al., 2013), OsPIP1;2 which is an aquaporin that improves yield (Xu et al., 2019), ABCG4 which is involved in abiotic stress responses (Matsuda et al., 2012), and OsPP2C30 which a core regulator in the ABA signaling pathway (Zong et al., 2016). Overall, our study supports that the TARGET assay is a fast and reliable approach to identify the direct TF→target genes in rice, bypassing the time-consuming process of developing transgenic rice. Importantly, the rapid rice TARGET TF-perturbation assay, can be used to prioritize rice TFs for more laborious studies *in planta*.

### Our network approach is transferrable to any phenotype in any organisms

The method we applied in this study relies on two inputs: a transcriptome-wide gene expression table and collected phenotypes from the same samples. With the reduced cost of RNA-Seq, especially with the 3′ RNA-sequencing (Weih, 2014; Groen et al., 2020; Weng and Juenger, 2022), it is much more feasible for researchers to obtain transcriptome expression data from many samples. Moreover, the software we used are all open-source and publicly available. This includes WGCNA (Langfelder and Horvath, 2008) for gene-to-phenotype correlation, GENIE3 (Huynh-Thu et al., 2010) for GRN inference and ConnecTF (Brooks et al., 2020) for network pruning. Putting these together, our network approach is not limited in rice research, but can be applied to any organism for any phenotype or trait.

## Conclusions

By using a combination of WGCNA and GENIE3 network methods, we present a gene regulatory network that links TF→target gene→NUEg phenotype to determine the mechanism of N-and/or-W signaling to the regulation of NUEg (**Figure 1**). We also show how to use TF-validation datasets from rice to validate inferred networks using ConnecTF (https://rice.connectf.org) (Brooks et al., 2020). In addition, we apply the cell-based TARGET temporal TF-perturbation system to rice to identify direct TF→target genes interactions and validate inferred gene networks. Overall, we identify a new role for OsbZIP23 and Oshox22 as regulators of the N-and/or-W signaling and regulation of NUEg, in addition to ABA/drought signaling. More broadly, we have identified 18 prioritized TFs and their targets that correlate with NUEg, and results from this network approach can potentially be used to optimize rice varieties to thrive in marginal low-N/arid soils, which are increasing in the face of global climate change.

## Data availability statement

The data presented in the study are deposited in NCBI repository, BioProject: PRJNA828338.

## Author contributions

CS, JH, C-YC, and GC designed the research experiments. CS, JH, C-YC, and H-JS, performed research experiments. JS, AH, MB, VA, and JA contributed data and analysis. CS, JH, C-YC, JS, AH, and GC wrote and edited the paper. All authors contributed to the article and approved the submitted version.

## Funding

This work is supported by NSF-PGRP IOS-1840761 to GC, a Grant from the Zegar Family Foundation (A16-0051) to GC, an NIH Grant RO1-GM121753 to GC, an NIH NIGMS Fellowship F32GM139299 to CS, and JS is an Open Philanthropy Awardee of the Life Sciences Research Foundation.

## Acknowledgments

We thank the staff at IRRI for their work on the field studies. We would also like to thank Dr. Manpreet Katari and Will Hinkley for their advice and sharing code for data analysis.

## Conflict of interest

The authors declare that the research was conducted in the absence of any commercial or financial relationships that could be construed as a potential conflict of interest.

## Publisher’s note

All claims expressed in this article are solely those of the authors and do not necessarily represent those of their affiliated organizations, or those of the publisher, the editors and the reviewers. Any product that may be evaluated in this article, or claim that may be made by its manufacturer, is not guaranteed or endorsed by the publisher.
