# Supplementary material for: Validation of a high-confidence regulatory network for gene-to-NUE phenotype in field-grown rice
Source: Front Plant Sci. 2022 Nov 25;13:1006044. doi: 10.3389/fpls.2022.1006044 (PMC9732682; doi:10.3389/fpls.2022.1006044)
Supplement: Supplementary file 11 [file Presentation_1.pdf]

Supplementary Material

1.1 Supplementary Figures

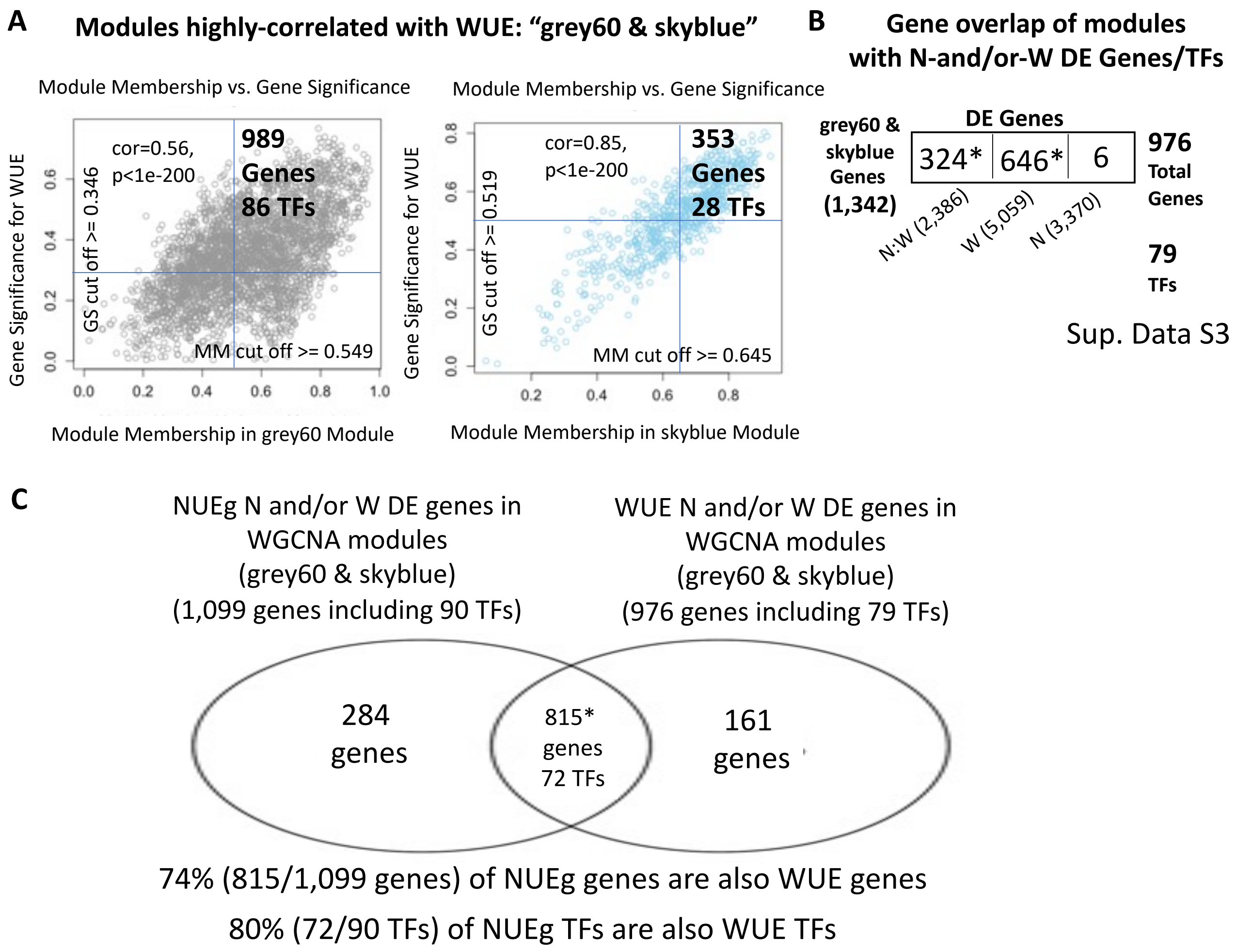

Supplementary Figure 1. WGCNA modules named “grey60” and “skyblue” are highly correlated with WUE. **(A)** Scatterplots of the WGCNA Gene Significance (GS) for WUE, versus the Module Membership (MM) for the grey60 and skyblue modules exhibit a significant correlation p-value < 0.001 with WUE. The genes with a GS and MM cut-off scores above the average score for the genes in each module were marked and selected for further analysis. **(B)** Significance of gene intersection (using Genesect in Virtual Plant 1.3 (Katari et al., 2010)) between the union of the genes and TFs with an above-average GS and MM score from the WGCNA grey60 and skyblue modules (grey60&skyblue) and the N:W, W, or N- responsive DE genes. Union of the genes in grey60 and skyblue modules: N-and/or-W response DE genes (324 + 646 + 6 = 976 genes) (Supplementary Data S3). **(C)** Significant overlap of the 1,099 NUEg N and/or W DE genes in WGCNA modules (grey60 & skyblue) with the 976 WUE N and/or W DE genes in WGCNA modules (grey60 & skyblue) p <

0.001 using Genesect in Virtual Plant 1.3. The overlap includes 815 genes and 72 TFs, and 16/18 candidate TFs from Table 1 (EIL4 and IDEF2 are not included).

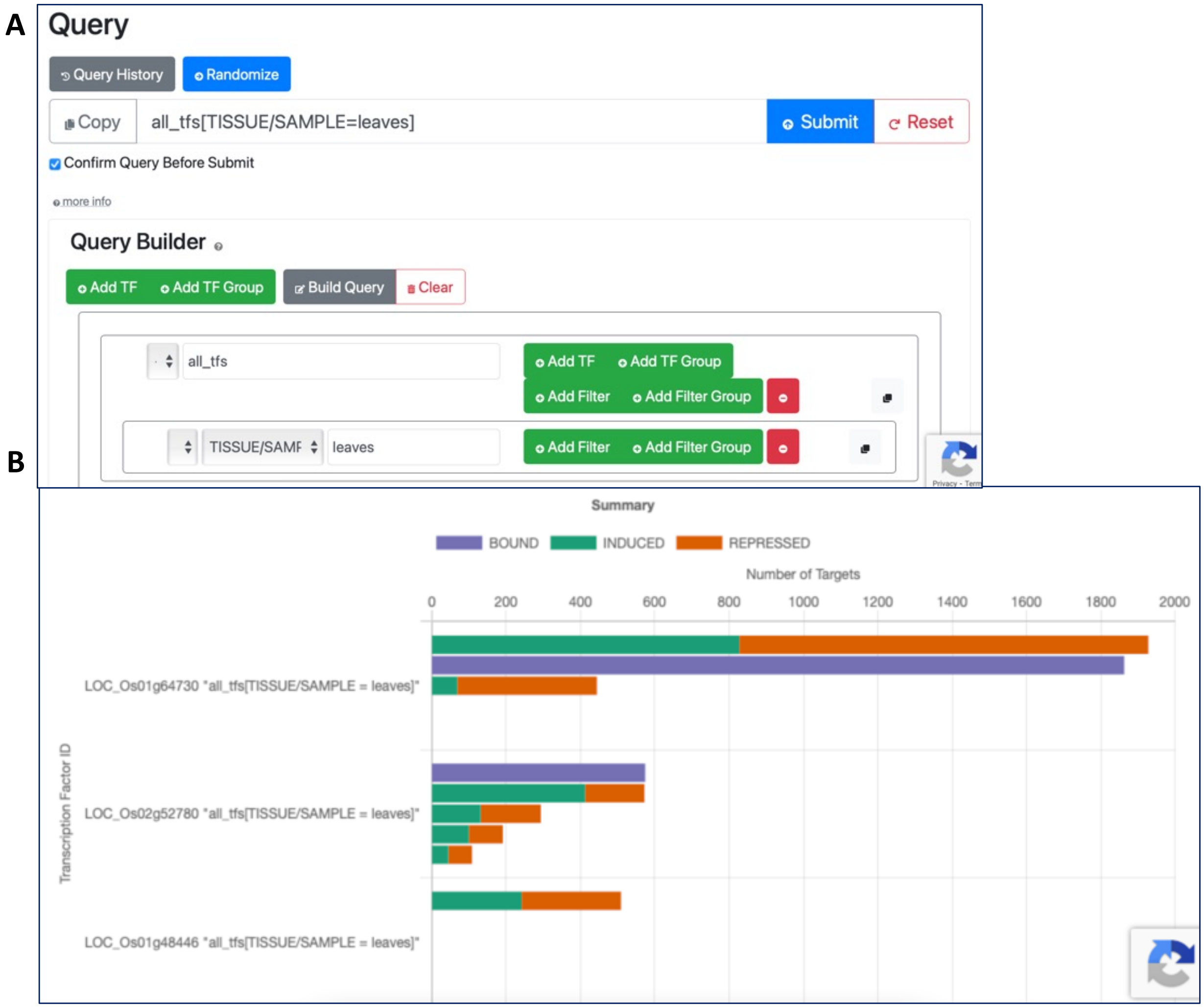

Supplementary Figure 2. ConnecTF platform query interface and summary for *in planta* validation data of the GENIE3 network. Screenshots from the query and summary pages from the GENIE3 network input in Connectf.org developed in Brooks et al., 2020 (Brooks et al., 2020). First, the total GENIE3 network for 90 TFs and 10,815 DE genes with 973,260 predicted edges (Supplementary Data S4) was uploaded to the “Target Network” pull down menu. **(A)** The ”Query Builder” was used to filter the ConnecTF database for all the data for TFs in the network and filtered for only leaf tissue. Pictured is screenshot of the query. **(B)** After the query is submitted the results ”Summary” (as pictured) is the first tab for the data found in ConnecTF. For the GENIE3 network, 9 datasets for 3 TFs were found in the network all form leaf tissue. These datasets include, *in planta* expression data RNA-seq, green bars induced genes and orange bars repressed genes) and bound data (ChIP-seq, purple bars) for three TFs, LOC_Os01g64730 (OsABF1) (Zhang et al., 2017), LOC_Os02g52780 (OsbZIP23) (Zong et al., 2016) and LOC_Os01g48446 (OsNAC14) (Shim et al., 2018). The data included, RNA-seq data transgenic overexpression rice lines for all three TFs, RNA-seq data for transgenic loss-of-function rice lines for OsbZIP23 and OsABF1, RNA-seq data for both

overexpression and loss-of-function lines for OsbZIP23 with a drought treatment, and finally ChIP-seq data for both OsABF1 and OsbZIP23. These datasets were then used for AUPR and network pruning in the network tab. Results shown in Figure 4.

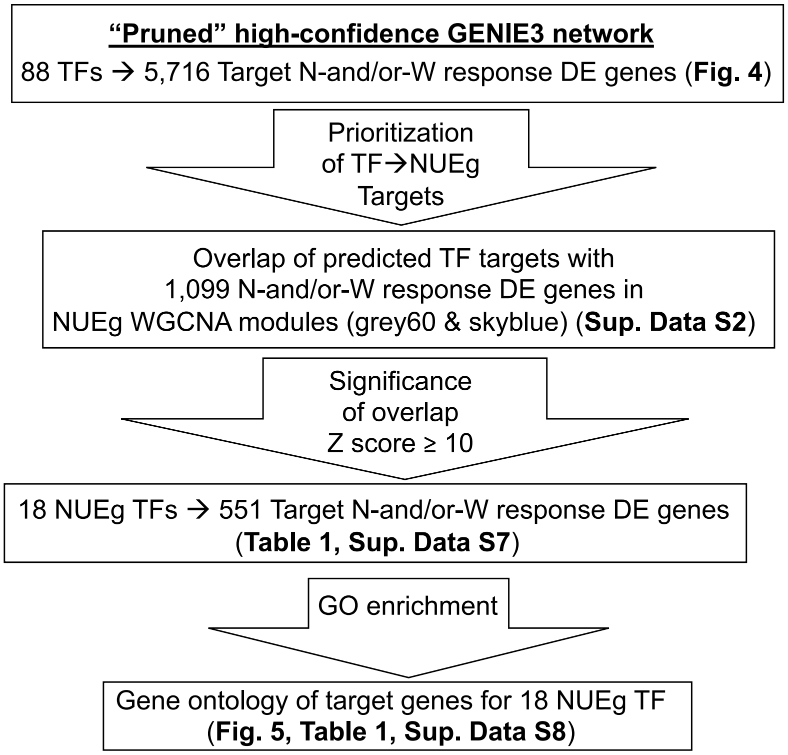

Supplementary Figure 3. Flow-chart for TF prioritization for the 88 TFs from the "pruned" GENIE3 network. The high-confidence TF→target genes for each of the 88 TFs were overlapped with the 1,099 N and/or W DE genes in the WGCNA modules (skyblue & grey60) associated with NUEg from Figure 3D using the Genesect function in Virtual Plant 1.3 (Katari et al., 2010). The 88 TFs were ranked based upon the Z-score for the overlap (Supplementary Data S6) and the TFs with the Z-score ≥ 10 were selected as candidate TFs. The full list of overlapping TF→target genes for these 18 TFs is in Supplemental Data S7 with a total of 551 target genes (Table 1). Next, Gene Ontology (GO) analysis for the overlapping target genes for each of the 18 candidate TFs (Table 1, Supplemental Data S8) was conducted with g:Profiler (Raudvere et al., 2019).

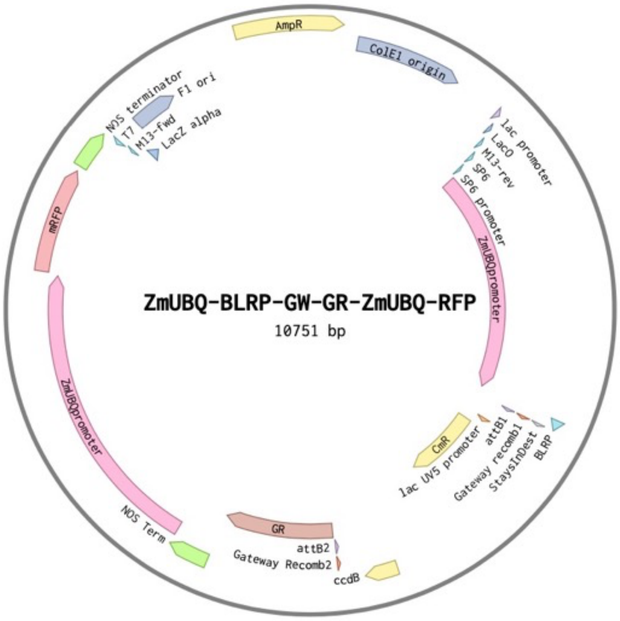

Supplemental Figure 4. Plasmid map for TARGET vector p1107 for TF-GR fusion protein expression. The sequence of the empty p1107 plasmid used for cloning is in Supplementary Data S10. This plasmid was used to express the TF-GR fusion protein for TARGET experiments. See materials and methods section for cloning methods.

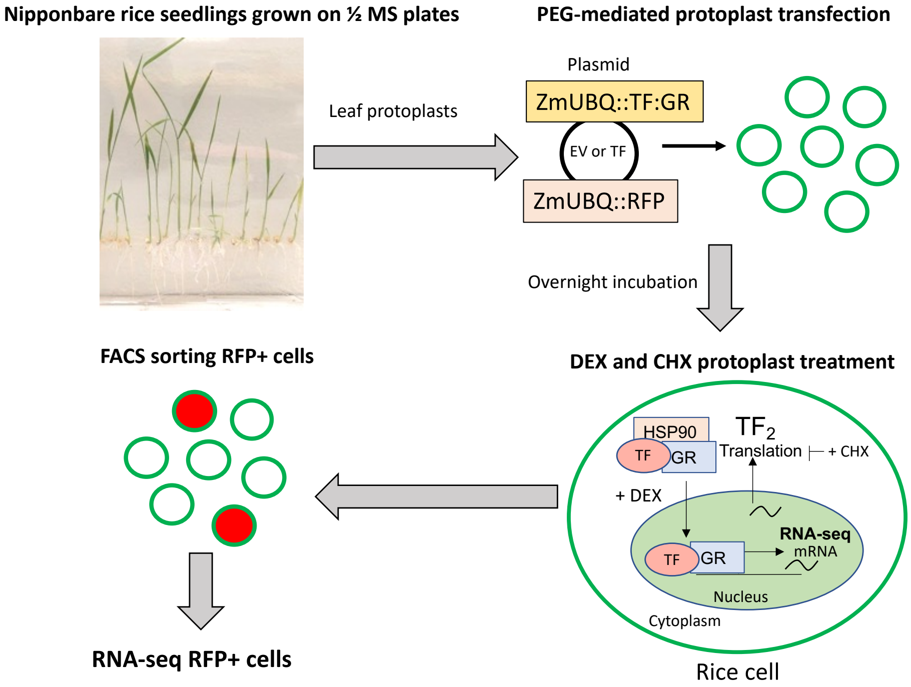

Supplementary Figure 5. The Transient Assay Reporting Genome-wide Effects of Transcription factors (TARGET) assay applied to rice leaf protoplasts. The schematic representation of the TARGET assay protocol adapted from Arabidopsis (Bargmann et al., 2013; Brooks et al., 2019).

Nipponbare seedling are grown on ½ MS plates for ~13 days. Protoplasts are isolated from leaf tissue and then transfected with the TF-GR fusion plasmid using polyethylene glycol (PEG). The transfected cells are incubated overnight and those with successful transfection will express the TF-GR fusion protein in addition to RFP. The TF-GR fusion protein is held in the cytoplasm by HSP90. Dexamethasone (DEX) treatment releases the TF into the nucleus to initiate transcription of direct target genes. Cycloheximide (CHX) treatment prevents translation of mRNA for secondary TF_2s and indirect TF→target gene responses. To amplify the TF→target gene expression data in RNA-seq, the protoplasts are FACS sorted for the RFP positive transfected cells before RNA-seq.

A **Genesect enrichment overlap between TARGET and *in planta* datasets**

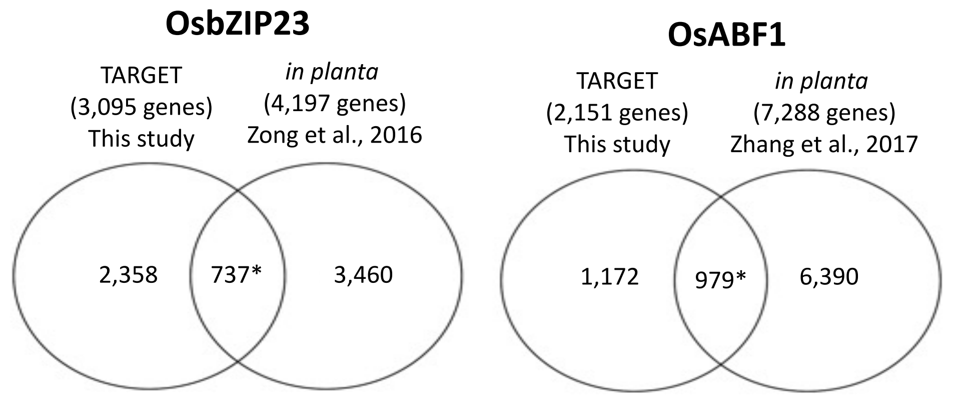

B **Validation of the pruned GENIE3 network with TARGET and *in planta* data**

TF Name	Data Type	Validated Edges	Predicted Edges	Validated Predictions	Precision	Precision %	Recall	Recall %	F-score
OsbZIP23	TARGET	3095	166	60	0.3614	36.1446	0.0194	1.9386	0.0368
OsABF1	TARGET	2151	258	35	0.1357	13.5659	0.0163	1.6272	0.0291
OsbZIP23	in planta	4197	166	52	0.3133	31.3253	0.0124	1.2390	0.0238
OsABF1	in planta	7369	258	90	0.3488	34.8837	0.0122	1.2213	0.0236

Ranked by F-score

Supplementary Figure 6. The TARGET assay is comparable to *in planta* data in identifying TF→target genes and validating the GENIE3 network. (A) The overlap of OsABF1 and OsbZIP23 between the direct TF→target genes from TARGET (Supplementary Data S9) and the combined *in planta* RNA-seq and ChIP-seq datasets downloaded from ConnecTF (Figure 4**,** Supplementary Figure 2) (Zong et al., 2016; Zhang et al., 2017). Genesect for p values and Z scores were determined with VirtualPlant 1.3 (Katari et al., 2010). Venn diagrams made with Venny 2.1.0. P value < 0.001 denoted with asterisk*. Z score for both overlaps is greater than 18. **(B)** The precision (rate of false positives), recall (rate of false negatives) and F-score (weighted average of precisions and recall) were calculated as in Brooks et al., 2019 (Brooks et al., 2019) to determine the performance of the pruned GENIE3 network at predicting the TF→target gene interactions.
